# Performance Analysis of Different Embedded Systems and Open-Source Optimization Packages Towards an Impulsive MPC Artificial Pancreas

**DOI:** 10.3389/fendo.2021.662348

**Published:** 2021-04-26

**Authors:** Jhon E. Goez-Mora, María F. Villa-Tamayo, Monica Vallejo, Pablo S. Rivadeneira

**Affiliations:** Grupo GITA, Facultad de Minas, Universidad Nacional de Colombia, Medellín, Colombia

**Keywords:** artificial pancreas, embedded control systems, model predictive control, optimization solver packages, type 1 diabetes

## Abstract

Current technological advances have brought closer to reality the project of a safe, portable, and efficient artificial pancreas for people with type 1 diabetes (T1D). Among the developed control strategies for T1D, model predictive control (MPC) has been emphasized in literature as a promising control for glucose regulation. However, these control strategies are commonly designed in a computer environment, regardless of the limitations of a portable device. In this paper, the performances of six embedded platforms and three open-source optimization solver algorithms are assessed for T1D treatment. Their advantages and limitations are clarified using four MPC formulations of increasing complexity and a hardware-in-the-loop methodology to evaluate glucose control in virtual adult subjects. The performance comparison includes the execution time, the difference concerning the evolution obtained in MATLAB, the processor temperature, energy consumption, time percentage in normoglycemia, and the number of hypo- and hyperglycemic events. Results show that Quadprog is the package that faithfully follows the results obtained with control strategies designed and tuned on a computer with the MATLAB software. In addition, the Raspberry Pi 3 and the Tinker Board S embedded systems present the appropriate characteristics to be implemented as portable devices in the artificial pancreas application according to the criteria set out in this work.

## Introduction

Closed-loop glucose control, referred to as artificial pancreas, has emerged as the best solution to modulate insulin doses in response to blood glucose (BG) concentration in subjects with type 1 diabetes (T1D). Artificial pancreas systems (APS) have been evaluated in clinical and home studies showing improved results than conventional sensor-augmented pump therapy. This extra-corporeal device consists of a continuous glucose monitoring (CGM) system that provides glucose measurements at regular intervals, a control algorithm that processes the CGM information and computes the appropriate insulin dosage, and an insulin infusion pump to execute the control action. All this process aims to emulate the natural behavior of the pancreas and provide a better quality of life to people with T1D (1, 2).

The control strategy is the core of APS. It coordinates the insulin delivery by the pump according to real-time CGM values. Numerous control strategies of varying complexity have been formulated for glucose control, as proportional-integral-derivative schemes (3, 4), model predictive control (MPC) formulations, and fuzzy logic (1). From these, MPC has received increasing attention due to its good performance in simulation and clinical tests (5, 6). Some MPC works in literature are the zone MPC (7) which incorporated the glycemia target as a set instead of a single point, some MPC designs with asymmetric cost function (8, 9), an MPC which drives glycemia to equilibrium sets and considers impulsive inputs (10), and an offset-free MPC with impulsive inputs that uses a disturbance model to compensate for a plant-model mismatch (11, 12). In addition, adaptive control strategies have been formulated as the MPC with adaptive penalization functions for matrices Q, R (13) and the impulsive offset-free strategy with adaptive features introduced in (14) and (15).

Although APS have been demonstrated to be effective in BG regulation, there is a need to design wearable devices to improve the quality of life of people with T1D. A crucial step towards a wearable APS is the implementation of control algorithms such as it performs on low-power and low-memory hardware without compromising the safety of the patient (16). Few approaches to control algorithm implementations have been reported. For instance, the Medtronic hybrid closed-loop system stands out, as it is the first commercially available device that operates to automatically compute basal doses while no meals are consumed (17). Also, control strategies implemented on a smartphone and evaluated on subjects with T1D can be found in (18–20), and a bihormonal wearable device with a custom-made printed circuit board and including a glucagon pump was tested in (21). Some embedded APS approaches were recently tested using hardware-in-the-loop (HIL) simulations using the cohort of virtual patients of the UVA/Padova simulator. In (22), an event triggered MPC was implemented in a Raspberry Pi 3B, and in (16) a periodic zone MPC was tested using an Arduino Feather M0. A complete review of APS architecture was presented in (2).

Additionally, the implementation of MPC strategies has been addressed from the point of view of the solver used for the intrinsic optimization problem in the control formulation. Active set methods, interior-point methods, and gradient projection methods stand out as families of solution algorithms for MPC (23). Variants of these methods have been developed as found in (16, 24–27). Under the APS framework, active-set and interior-point methods were tested with different open-source packages for C language: qsOASES, CVXGEN, ECOS and QPC in (2), primal-dual interior-point based on the predicator-correction algorithm was used in (28), a newton projection method was implemented in (29), and in (22) CVXOPT package for python was utilized.

In this work, an extended comparison of several available embedded systems is performed in terms of glucose regulation, computing processing unit (CPU) time, energy consumption, and temperature. The comparison is carried out by considering (i) four MPC formulations with increasing complexity; (ii) three different open source packages used for solving the optimization problem: CVXOPT, quadprog, and OSQP for Python; and (iii) six different embedded systems: Raspberry pi 3 model B (30), Raspberry pi 4 (31), Tinker Board S (32), Orange pi PC + (33), ODROID-XU4 (34) and Jetson Nano (35). Each combination of components (MPC strategy, solver, and embedded) is tested under the HIL simulation protocol and by considering scenarios with announced meals, sensor noise, and plant-model mismatch to induce hypoglycemia (BG < 70 mg/dl) and hyperglycemia (BG > 180 mg/dl). For each test, 10 adult virtual subjects are considered, whose parameters have been identified from the UVA/Padova simulator.

Additionally, simulations are performed to test the fidelity of results in embedded systems with respect to those obtained in Matlab. This with the aim of showing when an ARM processor architecture is capable of faithfully executing the computer-designed control algorithms. Moreover, the implementation of different control strategies allows discerning which type of optimization solver package is more suitable when the strategy’s complexity increases in terms of the number of decision variables and constraints.

The outline of the paper is as follows: Section II, III, and IV are disposed to briefly explain the four impulsive MPC strategies, three available toolboxes in python for solving the MPC optimization problem, and six embedded systems; Section V describes the simulation scenario and the metrics here considered, in Section VI the results of each simulation are presented; in Section VII the performance of the embedded systems and solver packages is discussed; in Section VIII the conclusions are exposed.

## Methods

### Model Predictive Control Strategies

In this section, a brief overview of impulsive MPC strategies for the artificial pancreas is provided. The impulsive scheme is selected knowing that insulin doses are administered as small spaced pulses rather than a continuous or a discrete input; therefore, it is appropriate to emulate the natural treatment of T1D. Here, the control-relevant model used for prediction in all MPC strategies is the one developed in (36), which has been discretized considering the impulsive form of the insulin delivery as in (37), and it results:

(1) $$\begin{array}{lll} x\left(k+1\right) & = & Ax\left(k\right)+B_{u}u\left(k\right)+B_{r}r\left(k\right)+E,\\ y\left(k\right) & = & Cx\left(k\right), \end{array}$$

where matrices are related with their continuous counterpart considering a fixed sampling time *T* as $A=e^{A_{c}T},\;B_{u}=e^{A_{c}T}B_{uc},\;B_{r}=\int_{0}^{T}e^{A_{c}s}dsB_{rc},\;E=e^{A_{c}T}E_{c},$ with:

(2) $$A_{c}=\left[\begin{array}{ccccc} -\theta_{0} & -\theta_{1} & 0 & \theta_{2} & 0\\ 0 & \frac{-1}{\theta_{4}} & \frac{1}{\theta_{4}} & 0 & 0\\ 0 & 0 & \frac{-1}{\theta_{4}} & 0 & 0\\ 0 & 0 & 0 & \frac{-1}{\theta_{5}} & \frac{1}{\theta_{5}}\\ 0 & 0 & 0 & 0 & \frac{-1}{\theta_{5}} \end{array}\right],$$

$$B_{uc}=\left[\begin{array}{c} 0\\ \begin{array}{c} 0\\ \begin{array}{c} \frac{1}{\theta_{4}}\\ 0 \end{array} \end{array}\\ 0 \end{array}\right],\;B_{rc}=\left[\begin{array}{c} 0\\ \begin{array}{c} 0\\ \begin{array}{c} 0\\ 0 \end{array} \end{array}\\ \frac{1}{\theta_{5}} \end{array}\right],\;E_{c}=\left[\begin{array}{c} \theta_{3}\\ \begin{array}{c} 0\\ \begin{array}{c} 0\\ 0 \end{array} \end{array}\\ 0 \end{array}\right]$$

and $C=\left[\begin{array}{ccccc} 1 & 0 & 0 & 0 & 0 \end{array}\right]$. The five state variables of the system are *x*_1_, the glycemia (mg/dl); *x*_2_ and *x*_3_, the delivery rates of insulin in the blood and interstitial space compartments, respectively (U/min); and *x*_4_ and *x*_5_, the delivery rates of carbohydrates in the stomach and gut compartments, respectively (g/min). The inputs are *u*, the exogenous insulin rate (U/min), and *r*, the carbohydrate intake rate (g/min). The output *y* corresponds to the glycemia. To implement the MPC formulations *via* state-feedback, a state estimator is required to obtain the complete state and reduce the noise of the BG measurement provided by a CGM sensor. In this work, the Kalman filter algorithm is implemented, but other approaches, as the moving horizon estimator, can be used.

In **Table 1**, four impulsive MPC formulations are reported. These have been previously tested in simulation scenarios *via* MATLAB for T1D treatment (11). The order in which they are reported in **Table 1** is according to their complexity, from the standard formulation to steer the state to a set-point to a more complex formulation that corrects plant-model mismatches and steers the state to an equilibrium target zone. All four strategies are initialized at each time step *k* with the initial estimated state $\hat{x}(k)$. The solution of each formulation is the optimal input trajectory ***u*** = {*u*(0), … , *u*(*H_c_* – 1}) and the predicted state trajectory ***x*** = {*x*(0), … , *x*(*H_p_* – 1)}. From ***u***, only the first element of the sequence is applied to the plant. When new measurement information is available, the optimization problem is reformulated and solved at the next time instant according to the receding horizon policy. The complexity of each MPC strategy can also be visualized in **Table 2** in terms of decision variables and the number of equality and inequality constraints.

**Table 1:** MPC strategies.

MPC strategy	Description	Optimization problem
Standard model predictive control (sMPC)	The sMPC aims to steer the system *x*, *u* to a set point *x_ref_*, *u_ref_*. It penalizes the deviation to the reference in the prediction and control horizons *H_p_* and *H_c_*, and it is subject to the dynamic constraint and the constraint sets *U*, *X*.	$\begin{array}{l} \underset{u,x}{\min}\sum_{j=0}^{H_{p}-1}||x\left(j\right)-x_{ref}{||}_{Q}^{2}+\sum_{j=0}^{H_{c}-1}||u\left(j\right)-u_{ref}{||}_{R}^{2}+||x\left(H_{p}\right)-x_{ref}{||}_{P}^{2}\\ \begin{array}{ll} s.t. & x(0)=\hat{x}(k)\\ & x(j+1)=Ax(j)+B_{u}u(j)+B_{r}r(j)+E\\ & u(j)\in \;U,\;x(j)\;\in \;X \end{array} \end{array}$
Zone model predictive control (ZMPC)	The ZMPC is formulated by adding a new decision variable *δ* and defining its upper and lower limits. This creates a zone such that, when the predicted variables are within it, the cost is zero.	$\begin{array}{l} \underset{u,x,\delta }{\min}\sum_{j=0}^{H_{p}-1}||x\left(j\right)-x_{ref}+\delta {||}_{Q}^{2}+\sum_{j=0}^{H_{c}-1}||u\left(j\right)-u_{ref}{||}_{R}^{2}+||x\left(H_{p}\right)-x_{ref}+\delta {||}_{P}^{2}\\ \begin{array}{ll} s.t. & x(0)=\hat{x}(k)\\ & x(j+1)=Ax(j)+B_{u}u(j)+B_{r}r(j)+E\\ & u(j)\in U,\;x(j)\in X\\ & \delta_{min}\leq \delta \leq \delta_{max} \end{array} \end{array}$
Zone model predictive control with artificial variables (ZMPC-AV) (37)	The ZMPC-AV introduces new decision variables *x_a_, u_a_* which are equilibriums of the system. The idea is to steer the system from an initial point to a point (*x_t_*, *u_t_*) in the target set $\left(X_{s}^{Tar},U_{s}^{Tar}\right)$ through the equilibrium of the impulsive system (*X_s_*, *U_s_*).	$\begin{array}{l} \underset{u,x,x_{a},u_{a},x_{t},u_{t}}{\min}\sum_{j=0}^{H_{p}-1}||x\left(j\right)-x_{a}{||}_{Q}^{2}+\sum_{j=0}^{H_{c}-1}||u\left(j\right)-u_{a}{||}_{R}^{2}\\ \;+P\left(dist_{X_{s}^{Tar}}\left(x_{a}\right)+dist_{U_{s}^{Tar}}\left(u_{a}\right)\right)\\ \begin{array}{ll} s.t. & x(0)=\hat{x}(k)\\ & x(j+1)=Ax(j)+B_{u}u(j)+B_{r}r(j)+E\\ & u(j)\in U,\;x(j)\in X\\ & x(H_{p})=x_{a}\\ & x_{a}=Ax_{a}+B_{u}u_{a}+E \end{array} \end{array}$
Offset-free zone model predictive control with artificial variables (ZMPC-AV-OF) (12)	This strategy compensates for the effect of a plant-model mismatch. To that end, the state is augmented with a disturbance *d*(*k*+1)=*d*(*k*), it is estimated with the state estimator, and then, this information is provided to the MPC problem in the prediction model and equilibrium constraints. The discrete model is now *x*(*k*+1)=*Ax*(*k*)+*B_u_u*(*k*)+*B_r_r*(*k*)+*B_d_d*(*k*)+*E*, *y*(*k*)=*Cx*(*k*)+*C_d_d*(*k*), with $B_{d}=e^{A_{c}T}B_{dc}$.	$\begin{array}{l} \underset{u,x,x_{a},u_{a},x_{t},u_{t},d}{\min}\sum_{j=0}^{H_{p}-1}||x\left(j\right)-x_{a}{||}_{Q}^{2}+\sum_{j=0}^{H_{c}-1}||u\left(j\right)-u_{a}{||}_{R}^{2}\\ \;+P\left(dist_{X_{s}^{Tar}}\left(x_{a}\right)+dist_{U_{s}^{Tar}}\left(u_{a}\right)\right)\\ \begin{array}{ll} s.t. & x(0)=\hat{x}(k),\;\;d(0)=\hat{d}(k)\\ & x(j+1)=Ax(j)+B_{u}u(j)+B_{r}r(j)+B_{d}d(j)+E\\ & d(j+1)=d(j)\\ & u(j)\in U,\;x(j)\in X\\ & x(H_{p})=x_{a}\\ & x_{a}=Ax_{a}+B_{u}u_{a}+B_{d}d+E,y_{a}=Cx_{a}+C_{d}d(j) \end{array} \end{array}$

**Table 2:** Complexity of the MPC strategies.

Strategy	decision variables	number of decision variables	number of inequality constraints	number of equality constraints
MPC	*u, x*	*H_c_*+*n_x_H_p_*	2*H_c_*+2*n_x_H_p_*	0
ZMPC	*u, δ, x*	*H_c_*+1+*n_x_H_p_*	2(*H_c_*+1)+2*n_x_H_p_*	0
ZMPC-AV	***u***, *x_a_*, *u_a,_ x_t,_ u_t,_* ***x***	*H_c_*+2*n_x_*+2+*n_x_H_p_*	2(*Hc*+2*n_x_*+2)+2*n_x_H_p_*	3*n_x_*
ZMPC-AV-OF	***u***, *x*_a_, *u_a_*, *x_t_*, *u_t_*, ***x***, ***d***	*H_c_*+2*n_x_*+2+(*n_x_*+*n_d_*)*H_p_*	2(*H_c_*+2*n_x_*+2)+2*n_x_H_p_*	3*n_x_*

H_c_, control horizon; H_p_, prediction horizon; n_x_, dimension of the state; n_d_, dimension of the disturbance considered in the ZMPC-AV-OF.

### Solvers for Optimization Problems

The most demanding part of an MPC strategy is the repetitive solution of the optimization problem, more strictly a quadratic problem (QP). Nevertheless, the efficiency of implementing an online MPC for embedded control applications depends on both the optimization problem and the selection of the optimization solver (2). For instance, power consumption can be reduced depending on solver runtimes. However, the optimality level can be altered by limiting the number of iterations performed by the solver.

There are several suitable software packages for real-time applications for solving the optimization problem in embedded systems. Here, only three open-source packages based on the Python programming language are considered:

#### Quadprog (Quadratic Programming)

The Quadprog solver uses the method developed by (38) for solving strictly convex quadratic programs. It is a dual active set method in which an iteration process is made in both primary and dual spaces, i.e., both the point and the Lagrange multipliers shift. The idea of the method is to obtain a primal optimal point for a sub-problem of the original problem, and then, a dual algorithm iterates to achieve primal feasibility that corresponds to dual optimality while assuring the primal optimality corresponding to dual feasibility. This is mainly accomplished by adding or removing constraints of the current active set at each iteration.

An advantage of this method is that a phase for finding a feasible point to start up the algorithm is not required since the unconstrained minimum of the problem is set as a starting point which can be considered as a near-optimal feasible point (as it is the optimal point of a subproblem). Besides, instead of directly using the pseudo-inverse of the normal vector of the constraints and an inverse Hessian operator for the quadratic *f*(*x*) subject to the active set, the Cholesky and QR factorization methods are used to obtain a numerically stable implementation of the algorithm.

#### CVXOPT (Python Software for Convex Optimization)

This package for convex optimization implements a standard Mehrotra predictor-corrector interior-point algorithm. It allows the user to specify an optimization problem *via* an operator description, i.e., by providing functions for evaluating the linear mappings in the constraints and to supply a custom method for solving the Newton equations.

#### OSQP (Operator Splitting Quadratic Program)

The OSQP solver is a numerical optimization package for solving convex QPs (39). It is based on a first-order alternating direction method of multipliers (ADMM), which can be seen as a variant of the alternating projections algorithm for finding a point in the intersection of two convex sets. ADMM has been shown to provide accurate solutions to QPs in a relatively small number of iterations. Hence, it is appropriate for embedded processors as it is computationally very cheap.

The OSQP solver uses an operator splitting technique with which no requirements on problem data as a positive definite cost function or linear independence of constraint are imposed. This solver only requires the problem to be convex. In fact, the splitting technique requires a single setup factorization as it is based on the solution of a quasi-definite linear system with coefficients that remain the same at almost every iteration. Therefore, after the setup, the algorithm is division-free, making it suitable for real-time applications in embedded systems

Additional characteristics of the OSQP solver are the detection of primal or dual infeasibility of the problem, the performance of *solution polishing* to obtain high accuracy solutions, the possibility of a warm start to reduce the number of iterations, a small compiled footprint of the code, among others. For further detail see (39, 40).

### Embedded Systems

For safety-critical applications, as disease treatment, HIL has been gradually considered as a required intermediate step between virtual simulations and complete physical prototyping (2). Therefore, the focus of this work relies on implementing the control algorithm in embedded systems and analyzing some challenges through HIL simulations. Six embedded systems were tested, whose characteristics are shown in **Table 3**. All processors have an ARM architecture that uses the reduced instruction set computer (RISC) system, where each command executes one instruction at a time, and the processor is divided into sectors for each type of assigned task, unlike x86 processors where compound commands are run, and the processors are general-purpose cores.

**Table 3:** Characteristics of Embedded Systems.

Embed	CPU	Frequency	RAM	storage	connectivity	source power	OS
Raspberry pi 4	Quad core Cortex-A72 (ARM v8) 64-bit	1.5 GHz	4 GB LPDDR4-3200 SDRAM	16 GB Micro SD	Gigabit Ethernet, BlueTooth 5.0	5V 3A	Raspbian
Raspberry pi 3 b	Quad Core Broadcom BCM2837 64bit	1.2 GHz	1GB LPDDR2 SDRAM	16 GB MicroSD	Ethernet, BlueTooth 4.0	5v 2.5A	Linux distributions, Windows
Tinker Board S	Rockchip RK3288 Cortex-A17 Quad Core 32-bit	1.8 GHz	2GB dual channel LPDDR3	16GB eMMC/MicroSD slot	Gigabit Ethernet, BlueTooth 4.0	5V/2-3A	Linux distributions, TinkerOS
Orange Pi s	Quad-core Cortex-A7 H.265 64-bit	1.6 GHz	2GB DDR3	8GB EMMC Flash/MicroSD	Ethernet, BlueTooth 4.0	5V 3A	Android Lubuntu, Debian, Raspbian
ODROID-XU4	Exynos5422 Cortex-A15 64-bit	2 GHz	2Gbyte LPDDR3	Flash 5.0, 16 GB MicroSD	Gigabit Ethernet	5V 4A	Linux distributions
Jetson Nano	Quad-core ARM A57	1.43 GHz	4 GB LPDDR4 25.6 GB/s	microSD	Gigabit Ethernet	5V 4A	Linux distributions NVIDIA Jetson software

The Tinker Board S is the only system that uses 32-bit architecture. However, as an extension of the ARMv7 architecture, this board implements the Advanced Single Instruction Multiple Data (SIMDv2) technology that allows supporting vector operations with integers and floating-points, and it also uses the Vector Floating-Point version 4 (VFPv4) for the calculation of the floating-point which is fully compatible with the IEEE-754 standard (41). The remaining embedded systems have processors of 64-bit ARM but with different configurations among them. The embedded system with the most potent processor architecture is the ODROID-XU4, with eight cores from which at least one reaches up to 2.0 GHz. Among the simplest embedded are the quad-cores of the Raspberry pi 3 and the Orange pi pc + with 1.2 and 1.6 GHz, respectively.

The embedded systems compared in this work use an operating system and are called simple boards (SB). The reasons for using simple-board systems are (i) the amount of memory, (ii) the versatility to use pre-compiled programming languages, (iii) they allow to host the three optimization packages and the four control strategies simultaneously, and (iv) they can be programmed to save and calculate statistics of the performance. The libraries, packages, and software used in this work are open source. This would reduce the equipment’s cost and increase acceptance of the open artificial pancreas project developed by the T1D community (https://openaps.org/). Although there are simpler and less energy-consuming embedded systems, it is better to have flexible electronic devices that can handle software’s updating, while algorithms, control strategies, and mathematical models for T1D treatment are in the testing phase.

For all embedded, the 16 GB class 10 microSD storage system is used, the corresponding Linux distribution and the software with the packages required to run the control algorithm are installed. For data transmission, the GPIO ports are configured for serial communication (RS232, Bd 115200, no parity, stopbits = 1, timeout = 90).

## Simulation Scenario and Metrics

**Figure 1:**
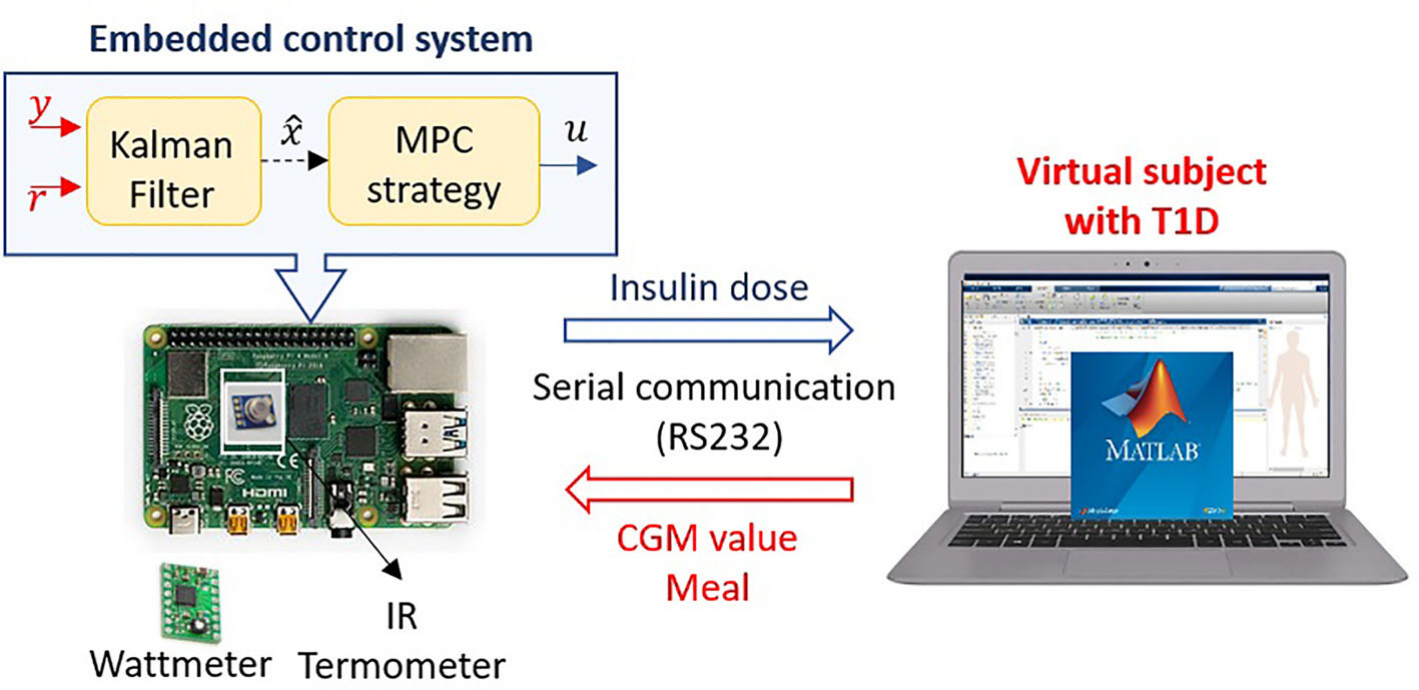
Hardware-in-the-loop implementation to emulate the AP system. The images used in this graphic are by unknown author under a CC BY-SA license. https://creativecommons.org/licenses/by-sa/3.0/.

The HIL technique is implemented to assess the six embedded systems’ performance and the three optimization solver packages with the four MPC strategies, as shown in **Figure 1**. The HIL is a real-time simulation between a computer that complies with the virtual representation of the plant and a real (hardware) version of the controller, which in this study corresponds to each embedded system that contains the MPC strategies and the estimator (42).

To represent the plant (the subject with T1D), model (1) is implemented in the computer using Matlab. As shown in previous studies, this model serves to emulate a virtual subject when considering proper scenarios (14, 15). To that extent, four aspects are taken into account:

1. Model (1) is personalized for 10 virtual adult subjects whose parameters have been identified to fit data from the commercial version of the UVA/Padova simulator (2014) (which is the first simulator approved by the FDA).
2. The parameters of the model are varied to emulate the net effect of physiological variations in the subject inducing hypoglycemia or hyperglycemia. To that aim, a 3-day simulation scenario is used in which the plant’s parameters are varied to induce hypoglycemia on day 1, there are no parameter variations on day 2, and the parameters are changed to induce a state of hyperglycemia in the subjects on day 3. Parameters are simultaneously varied by ± 10% of their nominal values (the ones identified to fit data), and this information is not provided to the controller.
3. Meals with different carbohydrate content are provided to the virtual subjects. The meals were set at 7, 10, 13, and 19-h with carbohydrate content of 55, 20, 90, and 70g, respectively. A duration of 15 min was considered for each meal, and meals were announced to the control strategies, i.e., this information is sent to the embedded system.
4. The sensor noise in the measurement is considered to use the CGM signal instead of glycemia. CGM signal has been studied based on Dexcom G5 mobile devices. Its model considers two linear polynomials to describe sensor calibration error, an additive measurement noise modeled by an autoregressive model that follows Gaussian distribution with zero mean and variance *σ* ^2^, and the interstitial glucose value (which corresponds to BG value with time delay). The equations describing the sensor error and their parameters can be seen in detail in (43).

Afterwards, the CGM signal is transmitted through a communication protocol (RS232) to the physical controller, where the calculations of the control actions are carried out as if they were working with an actual patient. The controller consists of a Kalman filter to estimate the state and reduce the noise effect, and an MPC strategy that finds the insulin dose that minimizes the corresponding optimization problem (according to **Table 1**). Next, at each time step, the amount of insulin to be delivered to the virtual patient is sent for the same communication protocol (RS232). This complete process facilitates the identification of the strengths, weaknesses, and challenges that must be faced before reaching a clinical study with real patients (44).

Regarding the set-up of simulations, the control parameters used for every strategy are: a prediction horizon *H_p_* = 80, a control horizon *H_c_*=10, a sampling time *T_s_*=5, and the initial state given by *X*_0_ = [115, *x*_2_ *_ss_*, *x*_3_ *_ss_*, 0, 0]*'*, where *x_2ss_* and *x_3ss_* refer to the equilibrium state of model (1) given by $x_{2ss}=x_{3ss}=\frac{\theta_{1}-115\theta_{2}}{\theta_{3}}$. For the sMPC, the reference was set as 95 mg/dl, and for the other three strategies, the target zone was set as [85-105] mg/dl. Weight matrices *Q, R*, and *P* were tuned in Matlab for each subject and then, these values were used in the tests of the embedded systems and optimization solver packages.

For the embedded systems, a Linux distribution was used with Python 3.x software. The optimization packages tested in the embedded are CVXOPT V1.2.6, Quadprog V0.1.8, and OSQP V0.6.2. The tolerances for each solver are reported in **Table 4** and can be modified by the exception of Python’s Quadprog package. The Python 3.x programming language uses the IEEE-754 standard for floating-point representation by default. Likewise, the embedded systems tested in this work have in their architecture modules compatible with the IEEE-754 standard. This standard uses a 64-bit floating-point format (double precision). Although 53 bits are used in this standard for the precision of calculated data, it should be noted that, in general, Python approaches the significant numbers up to 17 digits (45). Additionally, since small magnitudes are difficult to execute in an insulin injection mechanism, it was decided to round the result of the optimal insulin dosage at each instant *k* to handle minimum variations of 0.1 Units of insulin.

**Table 4:** Optimization package tolerances.

Package	Value of tolerances			
**Quadprog (MATLAB)**	TolPGG = 1e–5	Tolcon = 1e–4	TolX = 1e–4	Tolfun = 1e–4
**Quadprog (Python)**	N.A.	N.A.	N.A.	N.A.
**OSQP**	Eps_abs = 1e–3	Eps_rel = 1e–3		
**CVXOPT**	Feastol <1e–7	Abstol <1e–7	Reltol <1e–6	

For each test, the following data were saved: processing time per iteration, the total time of the simulation, delivered insulin, and blood glucose levels; and the following sensors were implemented: Gravity sensor - I2C Digital Wattmeter with a tolerance of ±0.2% to measure energy consumption, and the MLX90614 (GY - 906) sensor with a tolerance of ±0.5°C to measure processor temperature and the ambient temperature. The processor temperature was registered at rest and then stressed for 1 min. In addition, a performance reference was established as the one obtained in simulation with Matlab i.e., both the plant and the controller are running in Matlab, using Quadprog as the optimization algorithm. A computer Core i9-9880H with 16 GB RAM was used. The operating system was Windows 10, and the Matlab version was R2020a. This reference is used to compare the performances obtained in each test with the embedded systems.

Finally, a quantitative index was designed to select the embedded system that is more suitable for APS. The index is composed of seven criteria weighted according to their importance. The corresponding weights can be found in **Table 5**. This index is computed by maintaining the ZMPC-AV-OF strategy and the Quadprog package (with which the best results in glycemic control were obtained). Thus, for the selection of the best platform, the following criteria were considered:

- Percentage of time in range.
- The number of events above 180mg/dl.
- Coefficient of variation (CV) of blood glucose.
- Simulation time.
- Energy consumption.
- Temperature of the embedded device.
- The accuracy of the glycemic response obtained with each embedded system concerning the Matlab reference (measured with the mean absolute error).

**Table 5:** Overall performance evaluation for embedded system selection.

Criterion	Weight	Jetson Nano	ODROID-XU4	Orange Pi PC+	Raspberry Pi 3	Raspberry Pi 4	Tinker Board S
% Time in range	10,0	9,6	10,0	8,8	8,5	8,6	9,3
Events >180	10,0	9,6	9,4	9,6	9,4	9,4	10,0
CV	10,0	10,0	10,0	9,9	9,9	9,6	9,8
Simulation time	10,0	8,4	10,0	8,2	7,8	5,0	9,1
Energy consumption	20,0	9,6	7,9	13,6	20,0	6,0	12,5
Temperature	10,0	10,0	7,8	4,9	7,5	8,5	8,0
Accuracy w.r.t Matlab	30,0	28,3	28,0	28,5	28,6	27,6	30,0
**Total**	100,0	85,5	83,2	83,6	91,7	74,7	88,6

The last criterion has the highest weighing highlighting its importance (a weight of 30 was set). This term is directly related to this paper’s primary objective, which is evaluating the performance of the computer-designed controllers against their implementation in physical embedded systems. The second most important term was weighted with 20, and it is the energy consumption since the envisioned artificial pancreas will be implemented in a portable device with limited energy power.

The remaining factors are of equal importance and were set with a weight of 10. The terms (i)-(iii) can be assessed and improved in previous steps (i.e., in the designing phase) to the implementation of the control strategy in the embedded platform. The terms (iv) and (vi) are related to minor characteristics of the simple boards. The simulation time was considered minor since in a commercial insulin pump, the sampling time is 1 or 5 minutes, and from all the experimental tests, the maximum time obtained for one iteration was 6.9 seconds. Criterion (vi), although it affects the portable device’s performance, can be handled with heat sinks.

The weighting procedure for each embedded system consists of determining the platform with the best performance in each criterion and assigning to it the total weight of that term. The values for the remaining embedded systems are proportional to the difference with the best.

## Results

**Figure 2:**
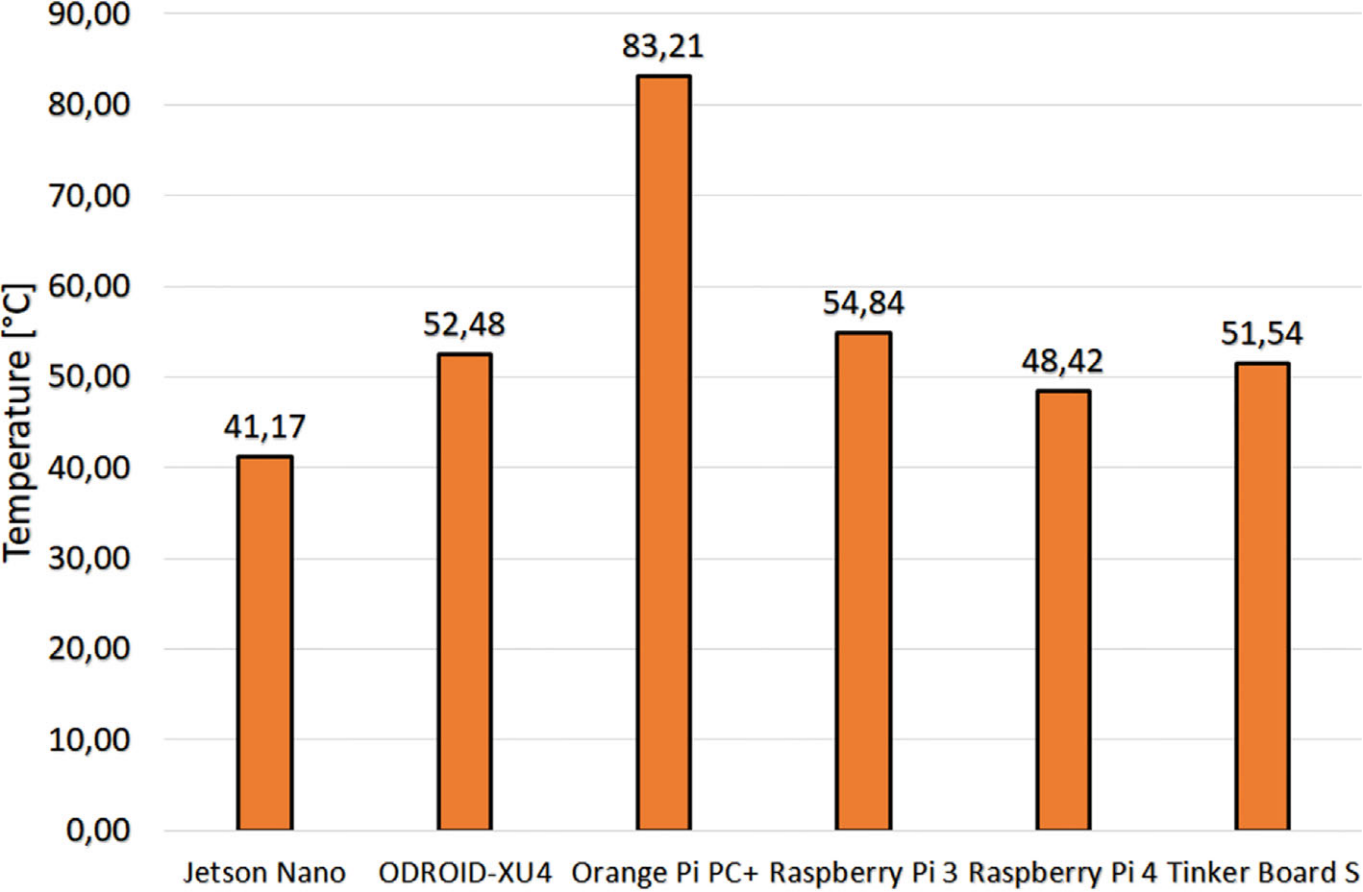
Temperature of the processor of each embedded system.

Temperature results of each embedded system when stressing their processors with the most complex strategy (the ZMPC-AV-OF) are shown in **Figure 2**. The embedded system with the lowest temperature increase when executing the code was the Jetson Nano registering 41°C. In contrast, the embedded system with the highest temperature was the Orange Pi pc + with 83°C. The average measurement of ambient temperature was 25°C, and the temperature at rest for all embedded systems was near 42°C.

**Figure 3:**
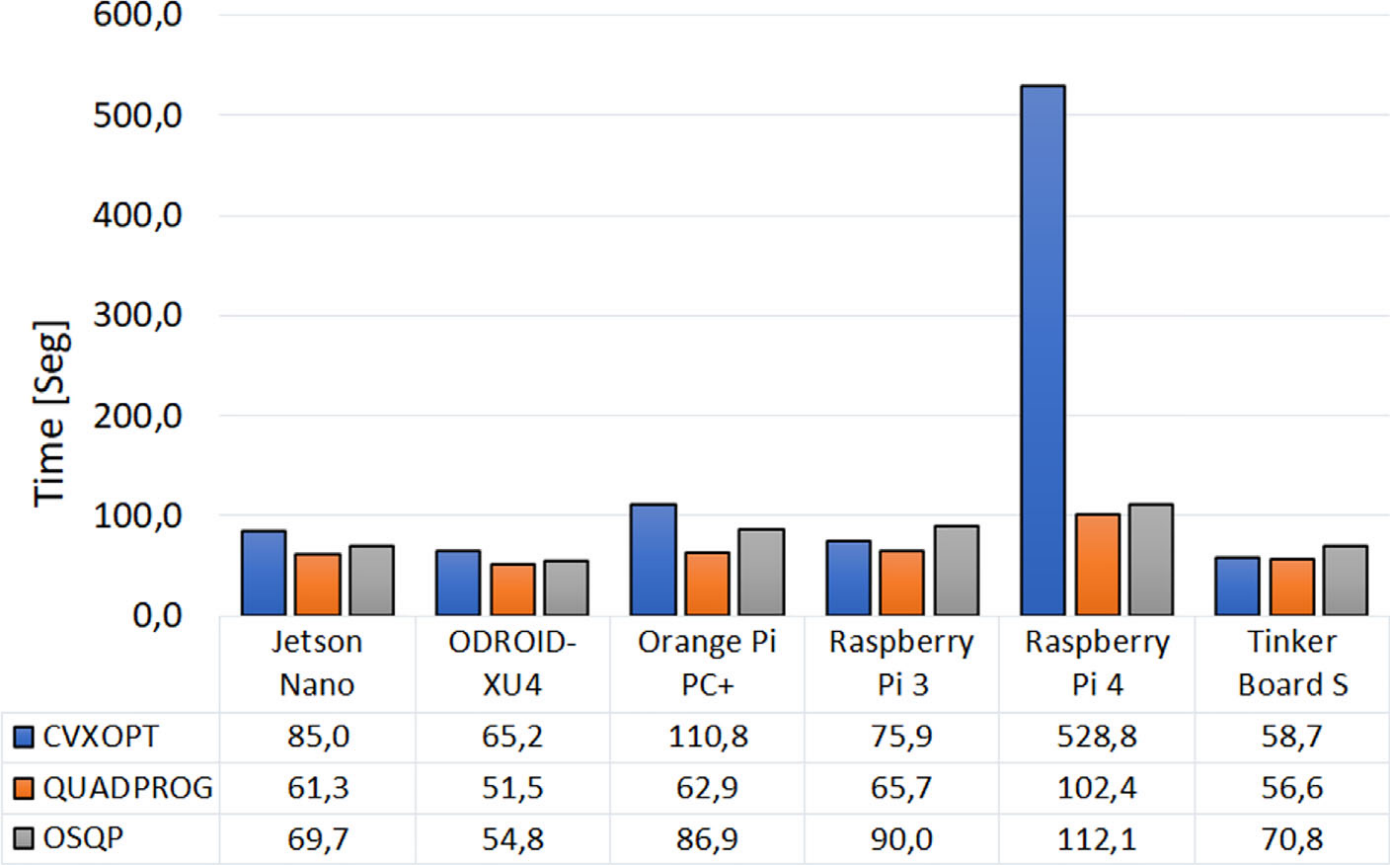
Average time to execute the four MPC strategies with the six embedded systems and per optimization solver package.

**Figure 3** depicts the average total computation time for executing the four MPC strategies using the three solver packages and the six embedded systems. The Quadprog package resulted in being the fastest package for any embedded device. Besides, from the embedded systems, the ODROID-XU4 presented the lowest total simulation time (51.5 s), followed by the Tinker Board S (56.62s). On the other hand, the Raspberry pi 4 presented the highest total simulation time with the three solver packages, especially when using the CVXOPT package, with which the total time was five times higher than in the other cases.

**Figure 4:**
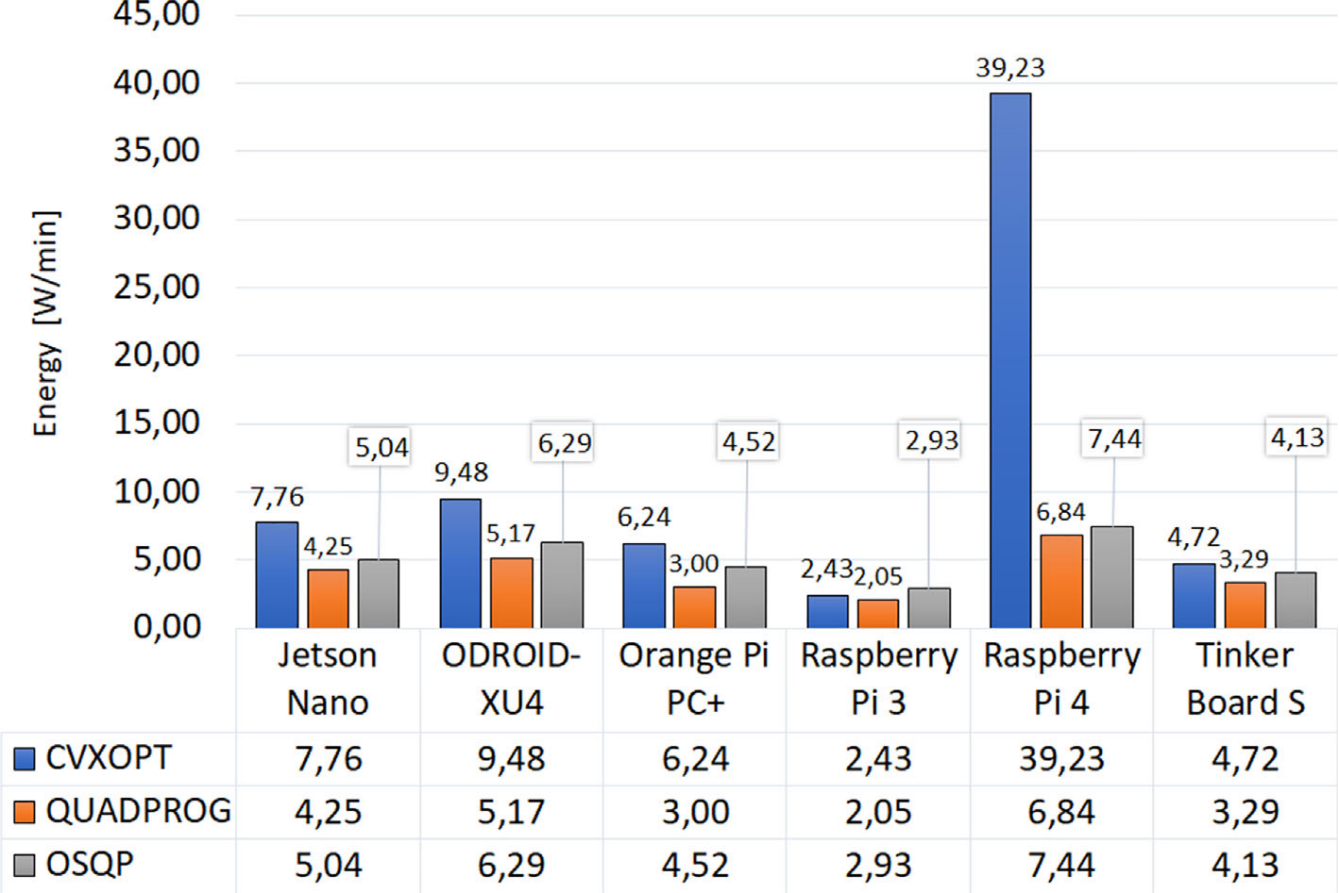
Consumed energy of each embedded system per package.

Energy consumption results are visualized in **Figure 4**. These are depicted as the average value of the energy consumed when executing the four MPC strategies. In most cases, there is a higher consumption linked to more complex strategies (ZMPC-AV and ZMPC-AV-OF). The Quadprog package generated the lowest consumption in all embedded systems, and the Raspberry Pi 3 stands out with an average consumption of 2.05 W/min, followed by the Orange Pi PC + with 3 W/min. Although the ODROID-XU4 presented the highest instantaneous power energy of all the embedded systems, it is not the one with the highest energy consumption due to the speed with which it executes the algorithm. The Raspberry pi 4 (especially with the CVXOPT package) was the highest energy consumption system because of the long simulation time.

**Figure 5:**
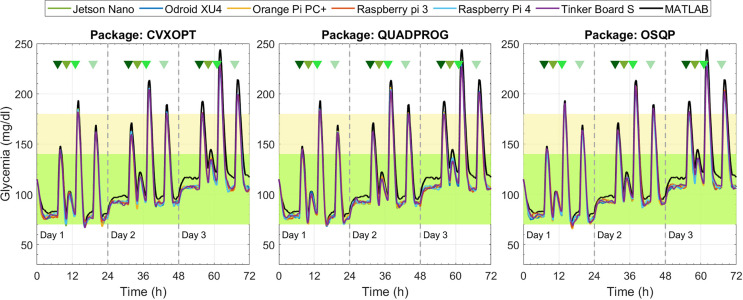
Glycemia evolution under the sMPC strategy with the three solver packages.

**Figure 6:**
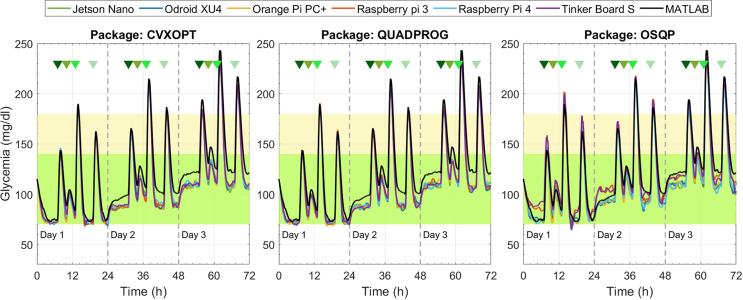
Glycemia evolution under the ZMPC strategy with the three solver packages.

**Figure 7:**
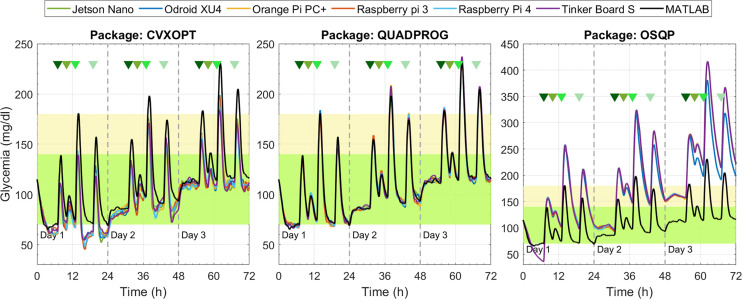
Glycemia evolution under the ZMPC-AV strategy with the three solver packages.

**Figure 8:**
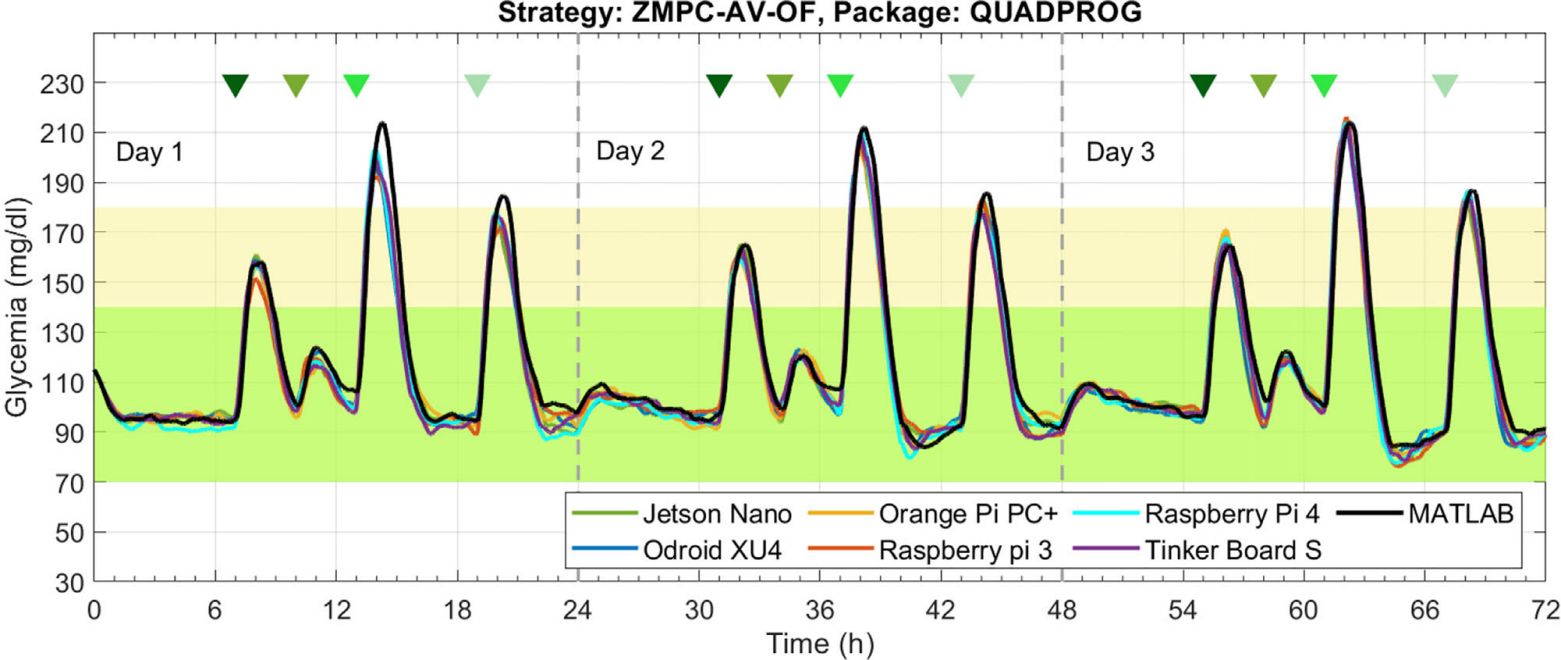
Glycemia evolution under the ZMPC-AV-OF strategy with the QUADPROG package in each embedded system.

From **Figures 5**–**8**, glycemia evolution controlled with the four MPC strategies is depicted for each embedded system and optimization solver package. Each figure shows the average level of blood glucose in the adult population. **Figures 5** and **6** show the system’s performance controlled by the sMPC and the ZMPC, respectively. As expected, their performances were affected by the parameter variations that induce hypo- and hyperglycemia in the plant. Thus, it is observed how BG levels decrease on day 1 and increase on day 3, evidencing an offset concerning the established target. In general, the glucose trajectories maintain the same trend, i.e., there are no significant variations between the different embedded systems (except for the ZMPC with the OSQP package). Nevertheless, it is observed that for long periods, the BG levels with the embedded systems remain below the BG levels obtained with Matlab (black lines).

**Figure 7** depicts the results under the ZMPC-AV strategy. For the OSQP and CVXOPT packages, the tolerances in **Table 4** were relaxed to obtain feasible solutions, i.e., to avoid the solver divergence and excessively high computational times (over 20 min per simulation). For the CVXOPT package, the tolerances were set as *Abstol=*3*e*-7, *Reltol=*4*e*-2, and *Feastol*=4*e*-2, and for the OSQP package as *Eps*_*abs*=90 y *Eps*_*rel*=9.9. With these two packages, the glycemia evolution controlled with the embedded systems varies with respect to the reference performance (obtained with Matlab). Hypoglycemic events occurred when using CVXOPT, and with the OSQP, control objectives were not achieved during the three days. In contrast, with the Quadprog package, the glycemia evolution controlled with any embedded system resulted very similar to that obtained with Matlab.

**Figure 9:**
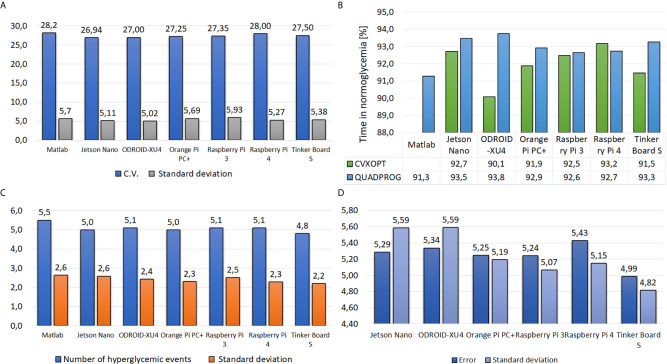
Results of each embedded system under the ZMPC-AV-OF strategy, **(A)** Coefficient of Variation, **(B)** Time percentage in normoglycemia, **(C)** Number of cases above 180 mg/dl, and **(D)** Error with respect to MATLAB.

The results obtained with the ZMPC-AV-OF strategy can be visualized in **Figures 8** and **9**. When using the Quadprog solver, the embedded systems’ results are very close to the Matlab reference, succeeding in avoiding hypoglycemia and reducing hyperglycemia events. Results obtained with CVXOPT and OSQP packages are not depicted because they generated the same issues illustrated with the ZMPC-AV strategy.

**Figure 9A** shows that a similar CV among subjects is obtained with all embedded systems. The CV remains under the 30%; therefore, it can be considered that glycemia does not vary significantly concerning the mean value of 213 mg/dl. **Figure 9B** depicts the time percentage in normoglycemia. In general, when using the Quadprog package, a higher time in normoglycemia is obtained; and when using the embedded systems Jetson Nano, ODROID-XU4, and Tinker Board S, a time over 93% resulted. Moreover, **Figure 9C**. refers to the number of events above 180 mg/dl. This figure shows that with the Tinker Board S, the lowest number of hyperglycemic events occurred (4.8 ± 2.4 events). Lastly, **Figure 9D** shows the average error of each simple board with respect to the glycemia evolution simulated in Matlab. Although this outcome is affected by the measurement noise, all the embedded systems have a good approximation to the reference (Matlab). The Tinker Board S stands out with the lowest average error of 4.99 mg/dl ± 4.82 mg/dl.

**Figure 10:**
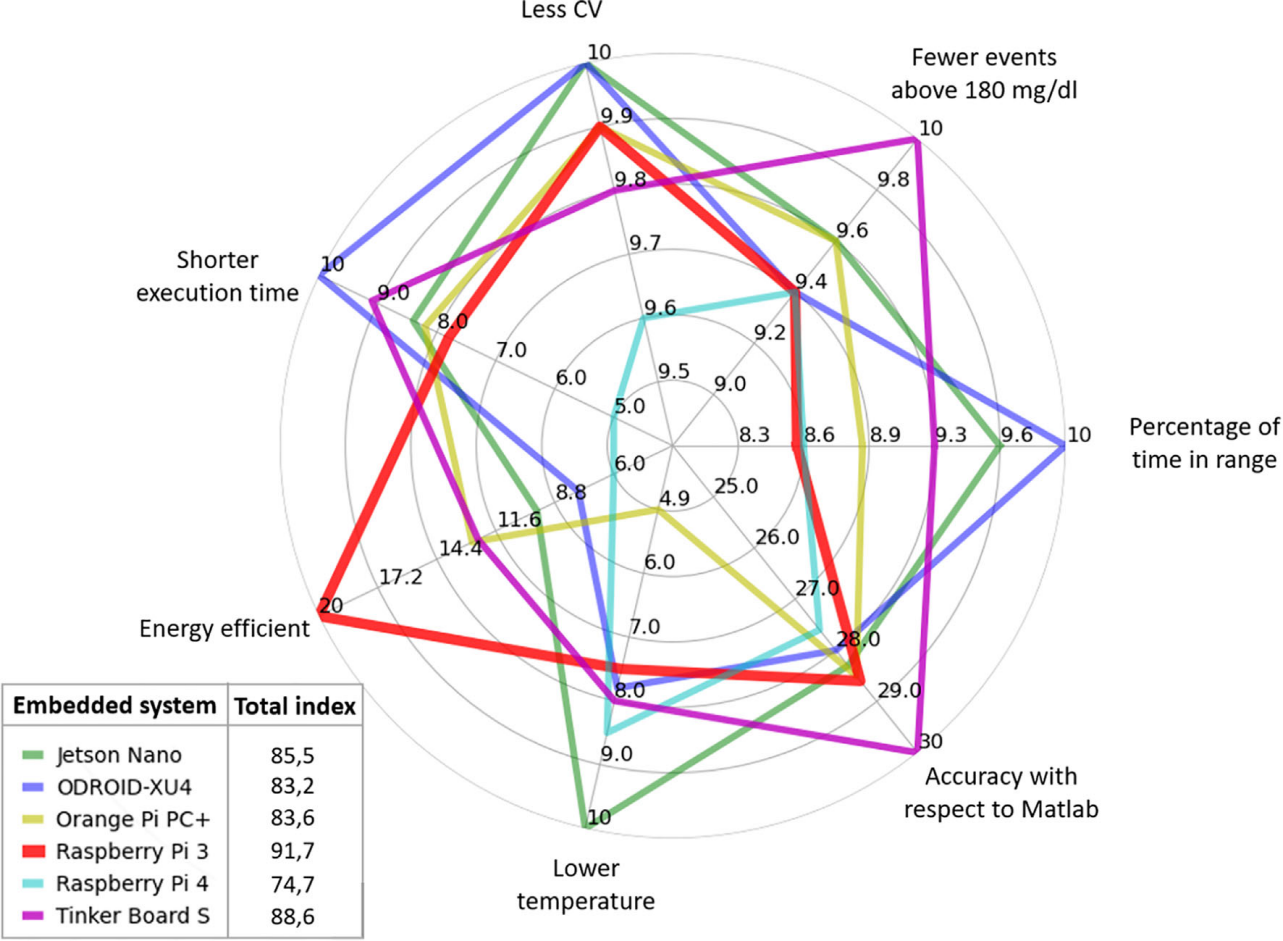
Weighting obtained by each embedded system in the performance criteria.

Finally, in **Figure 10**, the weighting obtained by each embedded system in each evaluated criterion is shown. For each criterion, the maximum assigned weight is depicted. The outer section of the plot indicates a better performance in the evaluated criterion. In contrast, the inner section of the spider plot is related to the less attractive measurements. For instance, recall that energy consumption has a maximum weight of 20, i.e., the embedded system with the lowest energy consumption (or most energy efficient) is assigned with a value of 20, which corresponds to the Raspberry Pi 3. The other simple boards are then assigned with a lower value in this criterion as they consume a higher amount of energy. The Raspberry Pi 4 is the board with the highest energy consumption and therefore with the lowest performance in the energy criterion, obtaining a value of 6. The assignment of weights to each platform is similarly done with the other criteria. In the end, the values obtained by each embedded system are added to find the best option.

The Total result of the summation shows that the Raspberry Pi 3 is the best option with a score of 91.7, followed by the Tinker Board S with 88.6. The Jetson Nano, the Orange Pi PC +, and the ODROID-XU4 obtained a score of 85.5, 83.6, and 83.2, respectively. The embedded system with the worst performance is the Raspberry Pi 4. The values of the weighting factors and the final performance index are reported in **Table 5**.

## Discussion

Regarding the temperature test, the Jetson Nano presented the lowest increase in temperature because it comes with a heatsink that spans much of the motherboard, which prevents heating the processor but makes it impractical for a portable device due to its size. Similarly, the ODROID-XU4 has a significantly large heatsink compared to the board’s size. In contrast, the high temperature obtained with the Orange pi pc + suggests that its operation would require a heatsink and possibly a fan, which is not desired for a portable device.

Although the hardware structure of the Raspberry Pi 4 has improved compared to the Raspberry Pi 3 (31), the higher energy consumption presented with this embedded system is not due to an increase in power when solving the optimization process. This energy consumption is more closely related to the algorithm’s execution time. From **Figure 3**, it can be seen that the time required to complete the simulation is substantially longer when implementing the CVXOPT package on the Raspberry Pi4 than with any other simple board, resulting in higher energy consumption, as seen in **Figure 4**. This behavior could suggest that there is no complete compatibility between the operating system, the programming language, or the optimization package used when conducting the experimental part of this study. Therefore, rather poor performance is obtained with long periods calculating the optimal insulin dose and high energy consumption.

A clear difference in processing times and energy consumption was obtained when running the complex control strategies (ZMPC-AV and ZMPC-AV-OF) compared to the simpler strategies (sMPC and ZMPC), which is expected. This difference is because of the higher computational cost with the complex strategies since the optimization problem to be solved at each time step has more constraints and more decision variables; therefore, the optimization problem to solve has higher dimensions (as evidenced in **Table 2**). However, the time per iteration is short compared with the sampling time (5 min) even for the most complex strategy, suggesting no impediment to implementing these MPC strategies in real-time. The results presented in (2) are comparable with the ones here obtained. When using the Raspberry pi 3 and the CVXOPT solver package for a ZMPC strategy in a single subject, the average time per iteration was reported as 50ms ±10ms, while here, the average time per iteration was 78ms ±17ms, yet, the prediction horizon used in (2) is much smaller (*H_p_*=20).

Regarding the control strategies’ performances with each solver package, a similar behavior among them was obtained with the simpler strategies: the sMPC and ZMPC. The results of **Figure 7** suggest that the OSQP package presents issues when increasing the complexity of the optimization problem (as shown for the ZMPC-AV and ZMPC-AV-OF strategies). For both control strategies, the virtual subjects cannot be kept within normal glucose ranges and substantially deviated from the Matlab results. This poor performance suggests an incompatibility between the OSQP solver and the more complex strategies, in which more decision variables and more equality and inequality constraints are added. The OSQP algorithm cannot find a suitable solution, even when the tolerances were significantly reduced. Other works corroborate this. For instance, in (16), where a ZMPC strategy was implemented, the fast alternating minimization algorithm (FAMA) was preferred to be used in embedded control systems because, in general, the ADMM algorithm presents limitations in the complexity bounds on the number of iterations and step size. Also, in (46), it is mentioned that the OSQP package presents an outstanding performance (comparable with commercial solvers) for small to medium-size problems.

A similar situation occurred with the CVXOPT package. With this package, satisfactory results are obtained when implementing the sMPC and ZMPC strategies. These good results were also obtained with a ZMPC formulation in (2, 22). Nevertheless, in neither paper, the CVXOPT package’s performance was tested with more complex optimization problems. The ZMPC used in (2, 22) has 4*H_c_*+2(*H_p_*-*Hu*) inequality constraints, while the ZMPC-AV explained in Section II has 2(*H_c_*+2*n_x_*+2) + 2*n_x_H_p_* inequality constraints plus the 3*n_x_* equality constraints due to the equilibrium artificial variables (*n_x_* refers to the dimension of the state). For this package and the ZMPC-AV-OF, it was possible to adjust the performance by modifying the tolerances to obtain feasible solutions. Nevertheless, very high sensitivity was presented in this adjustment, i.e., significant changes over glycemia were obtained for small changes of the tolerances, typically with a magnitude of 0.01, and especially in the gab relative tolerance. Besides, the same tolerances do not work well for all patients. These results suggest that the packages OSQP and CVXOPT are not reliable for T1D treatment, where the robustness and stability are of primal importance. Hence, both packages are considered invalid options for implementing an APS with complex control strategies.

Another factor that stands out from the results is that a complex and expensive architecture is not necessarily the best for all applications. For instance, the Jetson Nano has a GPU designed for parallel processing and focused on machine learning and digital image processing. However, these components are irrelevant for the application under study where the algorithm and its execution are sequential. Therefore, only the main processor’s performance is considered, which, although it presented good results, was not the best either in execution time or in performance. Thus, in this case, the Jetson Nano does not represent an advantage but an extra cost. The ODROID-XU4 presents a great instantaneous power consumption, which is compensated by its processing speed. Nevertheless, despite having the best processor specifications, this embedded system does not represent a substantial performance advantage over other simpler and cheaper embedded systems like the Tinker Board S or the Raspberry Pi 3.

On the other hand, the only embedded system with a 32-bit processor architecture is the Tinker Board S. However, The module added to the processor architecture (VFPv4) to compute the floating-point under the IEEE-754 standard (Double-precision) allows this embedded system not only to be compared with the other 64-bit platforms but also it presents an outstanding performance in many aspects evaluated in the tests. For example, this embedded system, together with the Raspberry Pi 3, manages a good balance between the energy consumed, the processing time, and the control objectives.

Regarding the data transmission in the results obtained, no significant differences were observed in the data transmission speeds *via* serial communication between each embedded system and the computer.

The outcomes also allow for assessing the level of confidence in the embedded systems when comparing them with the computer’s performance. There are vast differences between a computer and a simple board. The computer uses a processor x86 with the Complex Instruction Set Computer (CISC) architecture to run the algorithm by commands. In contrast, the embedded systems have an ARM processor that uses RISC. However, despite these differences, it could be seen that the results obtained in the computer (in Matlab) can be reproduced in the embedded systems when using the appropriate solver package. Therefore, when designing and tuning the control strategies in computers and then implementing them in the embedded systems, there is confidence that the embedded systems will execute the strategies adequately.

By considering the priorities for the T1D application and the results in each of the tests’ criteria, the Raspberry Pi 3 and the Tinker Board S turn out as the best options for APS when using advanced control strategies. It should be noted that, even though the Tinker Board S and other embedded systems have a better performance than the Raspberry Pi 3 in glucose regulation and algorithm execution times, the difference between them is not as significant as the low energy consumption presented by the Raspberry Pi 3. This last criterion is why Raspberry Pi 3 is located as the most appropriate for APS.

## Conclusions

In this work, the performance of six embedded systems and three open-source optimization packages were analyzed in terms of their implementation in the emulation of an APS using the HIL methodology. The embedded systems and solver packages were tested with four MPC strategies of increasing complexity. The results show that, when using the appropriate optimization package, ARM processors can successfully meet the goal of regulating glycemia in subjects with T1D even under complex control strategies. The Quadprog package resulted in being the best solver with faster and reliable responses concerning the other packages. The results obtained were very similar concerning those obtained in Matlab and gave guarantees of faithfully applying the algorithm in APS even though the optimization method used in the quadprog of MATLAB is different from the one available for python. In contrast, the OSQP and CVXOPT packages show difficulties in handling complex problems regarding the number of decision variables and constraints, making them unacceptable for T1D treatment.

Finally, the results obtained when using the more complex control strategy and the Quadprog package show no considerable difference in the embedded systems’ performance. However, a substantial difference is found in the energy consumed during the tests’ execution; thus, the embedded system recommended to be implemented as a portable device under the conditions exposed in this work is the Raspberry Pi 3 followed by the Tinker Board S.

## Data Availability Statement

The raw data supporting the conclusions of this article will be made available by the authors, without undue reservation.

## Author Contributions

JG-M developed the code of MPC strategies in Python, performed the tests with each embedded system, and drafted the manuscript. MV-T developed the code of the MPC strategies in Matlab and helped draft the manuscript. MV contributed with the theoretical framework of the study regarding embedded systems. PR conceived the study; he contributed with the study’s theoretical framework in impulsive control strategies and the analysis of the results. All authors contributed to the article and approved the submitted version.

## Funding

This work was supported by Minciencias (Colombia) with Grant 110180763081.

## Conflict of Interest

The authors declare that the research was conducted in the absence of any commercial or financial relationships that could be construed as a potential conflict of interest.
